# A desmoplakin variant associated with isolated arrhythmogenic left ventricular cardiomyopathy with rapid monomorphic ventricular tachycardia at first presentation

**DOI:** 10.1016/j.hrcr.2023.03.017

**Published:** 2023-03-28

**Authors:** Fu Guan, Thomas Wolber, Ardan M. Saguner, Argelia Medeiros, Oliver Müggler, Florian Berger, Matthias Gass, Nadine Molitor, Frank Ruschitzka, Corinna Brunckhorst, Firat Duru

**Affiliations:** ∗ Cardiac Arrhythmia and Electrophysiology Division, Department of Cardiology, University Heart Center, Zurich, Switzerland; † Center for Integrative Human Physiology, University of Zurich, Zurich, Switzerland; ‡ Swiss DNAlysis, Dübendorf, Switzerland; § Department of Cardiac Resonance Imaging, University Heart Center, Zurich, Switzerland

**Keywords:** Arrhythmogenic cardiomyopathy, Ventricular tachycardia, Desmoplakin, Genetic testing, Risk stratification

**Key Teaching Points**

• Arrhythmogenic right ventricular cardiomyopathy is the most typical form of arrhythmogenic cardiomyopathy (ACM), whereas biventricular ACM as well as left-dominant forms (arrhythmogenic left ventricular cardiomyopathy, ALVC) have been increasingly recognized. Mutations in the desmoplakin (*DSP*) gene were shown to be associated with ALVC.
• The present case reports a *DSP* c.1141-2A>T variant in a young female patient with ALVC who presents with ventricular tachyarrhythmia as first presentation.
• In addition to electrocardiography and imaging findings, genetic testing may serve as a key to avoid underdiagnosis of ALVC.

## Introduction

Arrhythmogenic cardiomyopathy (ACM) encompasses heart muscle diseases associated with potentially life-threatening ventricular tachyarrhythmias occurring out of proportion to the degree of underlying disease. The most classical disease is arrhythmogenic right ventricular cardiomyopathy (ARVC), whereas biventricular ACM as well as left-dominant forms (arrhythmogenic left ventricular cardiomyopathy, ALVC) have been increasingly recognized. Genetic variants in the desmoplakin (*DSP*) gene, coding for an integral part of the desmosome and the resultant disruption of intermediate filament binding, were shown to be associated with ACM, including ALVC.^1^ In this paper, we report a DSP variant associated with rapid sustained monomorphic ventricular tachycardia as first manifestation in a young female patient with isolated ALVC without right ventricle (RV) involvement.

## Case report

A 27-year-old White woman was hospitalized for presyncope associated with rapid monomorphic ventricular tachycardia with a heart rate of 260 beats/min following moderate consumption of alcohol. On admission, physical examination revealed a blood pressure of 70/40 mm Hg and an oxygen saturation of 95% in room air. Respiratory rate was 28 breaths per minute and no signs of pulmonary congestion were detected on auscultation of the lungs. Owing to the rapid heart rate, abnormal heart sounds were difficult to discern on cardiac auscultation. Electrical cardioversion was performed owing to hemodynamic instability. Laboratory tests showed elevated cardiac troponin with a maximum value of 262 ng/L (normal value <14 ng/L), which resolved to normal within 1 week, and considered to be related to acute myocardial injury owing to tachycardia. The chest radiogram on admission was normal. The patient reported 3 episodes of acute palpitations and dyspnea that occurred during the previous week, each lasting approximately 30 minutes prior to spontaneous termination. No signs of an inflammatory bout/myocarditis were present. She had no previous history of heart disease and was otherwise healthy. She performed regular personal training of moderate intensity twice a week. Her family background was not significant for sudden cardiac death, but the patient’s paternal grandmother had died abruptly at the age of 43 owing to an unclear heart condition. Her father also suffered from unclear cardiomyopathy since the age of 35, but had no accompanying arrhythmias. No further relevant family history could be obtained.

**Figure 1:**
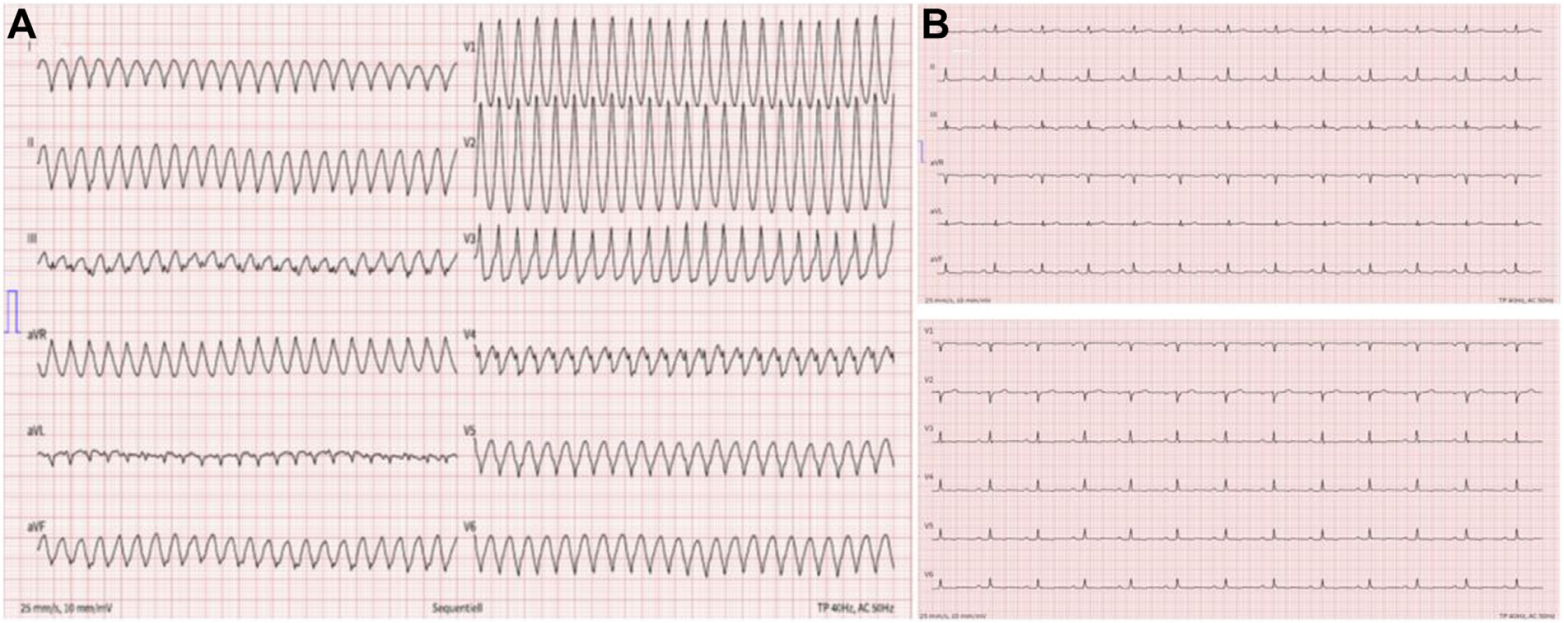
Electrocardiogram (ECG) findings. **A:** A 12-lead arrhythmia ECG: sustained monomorphic ventricular tachycardia with a right bundle branch block morphology, suggesting a left ventricular origin of the tachycardia. **B:** A 12-lead resting ECG. T-wave changes in inferior leads and precordial leads V3–V6, indicating left ventricular involvement.

**Figure 2:**
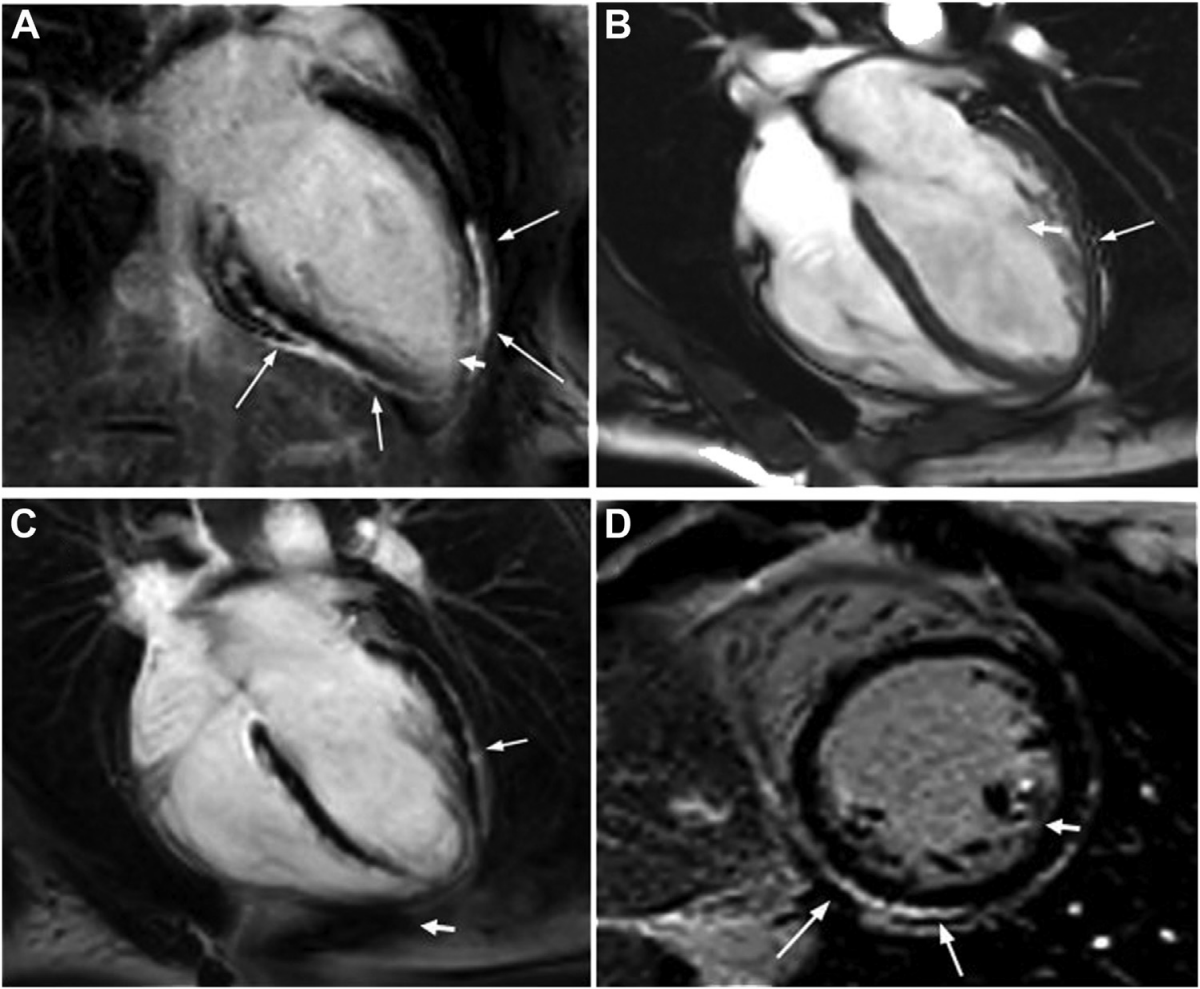
Magnetic resonance imaging (MRI) findings. **A, B:** Four-chamber long axis. **C:** Two-chamber view. **D:** Short-axis view. MRI shows normal left and right ventricular sizes. Panels A and B show fatty replacement within the left ventricular myocardium (white arrows). Panels C and D are left ventricular late gadolinium enhancement pattern, which is typical for arrhythmogenic cardiomyopathy (white arrows).

The 12-lead surface electrocardiogram (ECG) at presentation showed a rapid monomorphic wide-complex tachycardia with a right bundle branch block pattern and superior axis, suggesting ventricular tachycardia (VT) arising from an inferior left ventricular (LV) focus (Figure 1A). Resting 12-lead ECG after cardioversion in sinus rhythm was remarkable for T-wave negativity in inferior and left precordial leads (V_3_ through V_6_) and low QRS voltages (Figure 1B). Transthoracic echocardiography revealed normal LV systolic function (LV ejection fraction of 55%), but showed hypokinesia of the inferobasal and lateral apical segments of the LV. The level of hs-troponin I was elevated at 67.6 ng/L (normal <18 ng/mL) at admission and later reached a peak of 262 ng/L. C-reactive protein was normal, and no pericardial effusion was present, excluding perimyocarditis. A cardiac computerized tomography was subsequently performed, demonstrating normal coronary anatomy without calcifications and no significant coronary artery disease. Magnetic resonance imaging showed subepicardial late gadolinium enhancement of the inferior basal to apical as well as anterolateral midventricular to apical LV, partly with patterns suggestive of fatty replacement of the myocardium, well corresponding to the exit of the clinical VT, and revealed a normal RV. T1/T2-weighted images demonstrated an isointense signal; therefore an inflammation could be excluded. The size of both ventricles was within the normal range without akinetic or dyskinetic areas, whereas LV function was slightly reduced on magnetic resonance imaging (51%) (Figure 2). An invasive electrophysiological study was performed using a standardized protocol, which did not reveal any conduction disorder or inducible tachyarrhythmia. The ventricular stimulation protocol consisted of pacing at a basic cycle length at 600 ms and 400 ms, followed by up to 3 extrastimuli (S4) during pacing at 2 ventricular sites (right ventricular apex and right ventricular outflow tract), until reaching the refractory period or up to a minimum of 180 ms as shortest coupling interval between stimulations without and with intravenous isoprenaline, respectively. Isoprenaline was used as an induction agent up to a maximum dose of 8 micrograms/min, until the heart rate reached 200 beats/min).^2^^,^^3^ No ST-T changes were detected on the ECG during stimulation. Overall, the patient’s clinical profile was consistent with the diagnosis of ALVC.^4^^,^^5^

Genetic testing was performed with screening for variants in 44 cardiomyopathy-associated genes (*ABCC9, ACTA2, ACTC1, ACTN2, ANKRD1, BAG3, CSRP3, DES, DMD, DSC2, DSG2, DSP, EMD, FLNC, GATAD1, ILK, JUP, JPH2, LAMP2, LMNA, LDB3, MYBPC3, MYH6, MYH7, MYL2, MYL3, NEXN, NKX2-5, PKP2, PLN, PRDM16, PRKAG2, RBM20, SGCD, TCAP, TMEM43, TNNC1, TNNI3, TNNT2, TPM1, TTN, TTR, SCN5A*, and *VCL*) using the Swiss DNAlysis Cardiopanel (Agilent, Basel, Switzerand) with enrichment and sequencing with Illumina Miseq. A previously described heterozygous variant, c.1141-2A>T, was identified in intron 9 of the *DSP* gene. This variant has been previously described by Carruth and colleagues^6^ in a man with biventricular ACM, was well as by Shestak and colleagues^7^ in a patient with ARVC. However, this variant has not yet been described in a patient with isolated ALVC and ventricular tachyarrhythmia at first presentation.^8^^,^^9^ Other frameshift variants in the *DSP* gene have been reported to be associated with *DSP*-related diseases, including isolated ALVC.^10^ This particular variant is expected to affect the splicing of exon 10 of the *DSP* gene, with unknown consequences. According to the American College of Medical Genetics and Genomics criteria, the *DSP* c.1141-2A>T variant is classified as likely pathogenic (PVS1, PM2) based on the current state of knowledge. The patient underwent implantation of a VDD transvenous implantable cardioverter-defibrillator and was discharged on bisoprolol 2.5 mg per os per day. In addition, she was advised to limit her exercise activity. After 4 months, she experienced 1 appropriate implantable cardioverter-defibrillator shock owing to fast monomorphic VT during exercise after having forgotten to take her beta-blocker on that day. Six months follow-up shows that she is doing well, without any further sustained ventricular arrhythmias.

## Discussion

DSP is one of the main proteins of the desmosome linking the cytoskeletal networks to the cellular membrane, particularly connecting the desmocollins and desmogleins by interacting with plakoglobin and plakophilin and with the intermediate filaments. Basso and colleagues^11^ were first to demonstrate that ACM arises secondary to genetic variants in the *DSP* gene. Later, it was found that *DSP* variants were associated with the pathogenesis of ALVC.^12^ Of note, 75% of this population had VT as the primary clinical manifestation, which was previously under-recognized.^4^ Some human *DSP* gene variants are associated with ACM, which manifest as early isolated LV disease or prior to RV disease.^13^^,^^14^ In this report, we present a DSP variant in a patient with isolated ALVC with tachyarrhythmia as first manifestation. This finding may contribute to improving the diagnosis of ALVC presenting with life-threatening ventricular arrhythmias.

In summary, variants of DSP are associated with ARVC, ACM, and ALVC. We demonstrated that the DSP c.1141-2A>T variant is associated with ALVC, with a life-threatening arrhythmia being the first manifestation. We concluded that in addition to ECG and imaging modalities, it is of paramount importance to perform genetic testing in order to avoid underdiagnosis.
